# Acid Stability of the Hemagglutinin Protein Regulates H5N1 Influenza Virus Pathogenicity

**DOI:** 10.1371/journal.ppat.1002398

**Published:** 2011-12-01

**Authors:** Rebecca M. DuBois, Hassan Zaraket, Muralidhar Reddivari, Richard J. Heath, Stephen W. White, Charles J. Russell

**Affiliations:** 1 Department of Structural Biology, St. Jude Children's Research Hospital, Memphis, Tennessee United States of America; 2 Department of Infectious Diseases, St. Jude Children's Research Hospital, Memphis, Tennessee, United States of America; 3 Department of Microbiology, Immunology & Biochemistry, University of Tennessee Health Science Center, Memphis, Tennessee, United States of America; Institut Pasteur, France

## Abstract

Highly pathogenic avian influenza viruses of the H5N1 subtype continue to threaten agriculture and human health. Here, we use biochemistry and x-ray crystallography to reveal how amino-acid variations in the hemagglutinin (HA) protein contribute to the pathogenicity of H5N1 influenza virus in chickens. HA proteins from highly pathogenic (HP) A/chicken/Hong Kong/YU562/2001 and moderately pathogenic (MP) A/goose/Hong Kong/437-10/1999 isolates of H5N1 were found to be expressed and cleaved in similar amounts, and both proteins had similar receptor-binding properties. However, amino-acid variations at positions 104 and 115 in the vestigial esterase sub-domain of the HA1 receptor-binding domain (RBD) were found to modulate the pH of HA activation such that the HP and MP HA proteins are activated for membrane fusion at pH 5.7 and 5.3, respectively. In general, an increase in H5N1 pathogenicity in chickens was found to correlate with an increase in the pH of HA activation for mutant and chimeric HA proteins in the observed range of pH 5.2 to 6.0. We determined a crystal structure of the MP HA protein at 2.50 Å resolution and two structures of HP HA at 2.95 and 3.10 Å resolution. Residues 104 and 115 that modulate the acid stability of the HA protein are situated at the N- and C-termini of the 110-helix in the vestigial esterase sub-domain, which interacts with the B loop of the HA2 stalk domain. Interactions between the 110-helix and the stalk domain appear to be important in regulating HA protein acid stability, which in turn modulates influenza virus replication and pathogenesis. Overall, an optimal activation pH of the HA protein is found to be necessary for high pathogenicity by H5N1 influenza virus in avian species.

## Author Summary

To deliver their genomes into host cells during entry, enveloped viruses contain glycoproteins that bind to cellular receptors and cause fusion of viral and cellular membranes. The influenza virus HA protein is the archetypal viral fusion glycoprotein, promoting entry by undergoing irreversible structural changes that drive membrane merger. HA trimers on the surfaces of infectious influenza virions are trapped in a metastable, high-energy conformation and are triggered to refold and cause membrane fusion after the virus is internalized and exposed to low pH. Here, we provide biochemical and x-ray crystallographic evidence that naturally occurring amino-acid variations at the interface of the vestigial esterase and fusogenic stalk domains alter HA acid stability for highly pathogenic H5N1 influenza, resulting in a shift in the threshold pH required to activate HA protein structural changes that cause membrane fusion. Furthermore, our data reveals that an increased HA activation pH correlates with increased H5N1 virulence in chickens. Overall, the acid stability of the HA protein is identified as a novel virulence factor for emerging H5N1 influenza viruses. A major implication of this work is that the fitness of enveloped viruses may be fine-tuned by mutations that alter the activation energy thresholds of their fusion glycoproteins.

## Introduction

Highly pathogenic avian influenza (HPAI) viruses kill up to 100% of infected poultry flocks and may cause high mortality rates when transmitted to humans [1], [2]. For example, H5N1 influenza viruses have contributed to the deaths of 331 of 565 individuals since 2003 [3] and are endemic in domestic poultry in Egypt and Indonesia [4]. The continued circulation of H5N1 and potential emergence of an H5N1 human pandemic virus remain ever-present threats.

The hemagglutinin (HA) surface glycoprotein promotes viral entry through its receptor binding and membrane fusion functions [5], and mutations in HA have been shown to modulate the pathogenicity, host range specificity, transmissibility, and pandemic potential of influenza viruses [1], [6], [7]. HA is synthesized as a trimeric HA0 protein that must be activated for membrane fusion by post-translational cleavage into a high-energy HA1/HA2 complex. The multi-basic HA0 cleavage sites of H5 and H7 HPAI viruses are recognized by ubiquitously expressed intracellular proteases, facilitating systemic virus spread and greater pathogenicity [8]–[10]. HA binds to sialic acid-containing receptors on the surfaces of host cells [5], and the specificity of receptor binding helps determine host range, with avian and human viruses preferentially binding to α(2,3) and α(2,6) sialosides, respectively [11], [12]. Upon internalization, the virus is exposed to progressively lower pH values until a threshold is reached that triggers HA to undergo irreversible conformational changes that mediate membrane fusion [13]. Mutations that modulate HA acid stability have been associated with the adaptation of influenza viruses to different host species and cell lines [14], [15], and HA acid stability has recently been identified as a potential virulence factor [16].

Some influenza viruses contain all of the known genetic elements for high pathogenicity yet are attenuated *in vivo*. For example, the clade 3 H5N1 isolate A/goose/Hong Kong/437-10/1999 has significantly lower replication and pathogenicity in chickens compared to the closely related isolate A/chicken/Hong Kong/YU562/2001 [17]. The attenuating amino-acid residues have been mapped to the receptor-binding sub-domain and the vestigial esterase sub-domain in the HA1 receptor-binding domain (RBD) in the HA protein. However, the HA proteins from both isolates contain markers typical of high pathogenicity including a polybasic cleavage site, identical glycosylation sites, and identical residues in the receptor-binding pocket. The goal of the current study was to determine the molecular mechanism by which the naturally occurring variations in the HA protein modulate H5N1 pathogenicity.

## Results

### MP and HP HA proteins have different acid stabilities

**Figure 1:**
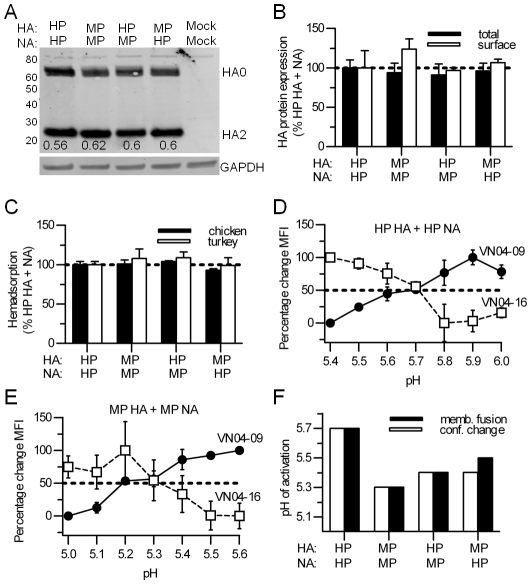
Characterization of the HP and MP HA proteins. (**A**) Western blot of HA expression. Values below the HA2 bands are the cleavage ratio, calculated by dividing HA2 by total HA (i.e., HA0 + HA2). The values listed are the mean of 3 independent experiments. (**B**) HA protein expression. Total expression (solid bars) was measured by Western blot and surface expression (open bars) was measured by flow cytometry. Values were normalized to 100 % for HP HA (+ HP NA). (**C**) Hemadsorption of chicken and turkey erythrocytes to cell surface-expressed HA proteins. Bound erythrocytes were lysed, and released hemoglobin was measured at 415 nm. Values were normalized to 100% for HP HA (+ HP NA). (**D** and **E**) pH of HA protein conformational changes. HP and MP HA proteins were co-expressed with their homologous NA proteins. Activation pH values for HA proteins were measured by flow cytometry using conformation specific monoclonal antibodies Vn04-09 and Vn04-16, that preferentially recognize prefusion and acid-activated forms of the H5N1 HA protein, respectively. (**F**) Activation pH of the HA protein, expressed as the midpoint of the pH of conformational changes (closed bars) and the highest pH at which syncytia formation occurs (open bars). Error bars in panels **B, C, and F** represent the standard deviation of triplicate experiments.

To determine how the HA proteins from the two isolates differ in their biochemical properties, the proteins were expressed in cell culture and compared for expression, cleavage, receptor binding, and activation pH (Figure 1, S1). The neuraminidase (NA) proteins were co-expressed with the HA proteins because of the known interplay between HA and NA with respect to receptor binding [18] and membrane fusion [16], [19], [20]. Western blot and flow cytometric analyses revealed no significant differences in total or cell-surface expression of the two HAs when co-expressed with NA from either isolate (Figure 1A,B and Figure S1). The ratios of cleaved (HA1+HA2) to uncleaved (HA0) species were also similar for the two HAs (Figure 1A). To investigate potential differences in avian receptor-binding avidity, we quantified the amount of chicken and turkey erythrocytes adsorbed to HA-expressing cells and found no difference (Figure 1C). Overall, these data show that the amino-acid variations in the HA and NA proteins from the MP and HP isolates do not result in substantial differences in HA protein expression, cleavage, or receptor binding.

We next compared the activation pH values of the two HA proteins. Flow cytometry was used to measure pH-induced conformational changes by using monoclonal antibodies VN04-09 and VN04-16 that preferentially bind to the prefusion and postfusion forms of the H5N1 HA protein, respectively [19], [21]. When co-expressed with the homotypic NA partner, conformational changes by MP HA were first observed at pH 5.4, reached a midpoint at pH 5.3, and were complete at pH 5.1–5.2 depending on the antibody (Figure 1E). In contrast, conformational changes by HP HA were triggered at higher pH (∼0.4 pH units) being first observed at pH 5.8, reaching a midpoint at pH 5.7, and being complete at approximately pH 5.4 (Figure 1D). Comparing the midpoints of conformational changes, MP HA had a value of pH 5.3 whereas HP HA had a value of 5.7, showing that HP HA is less acid stabile than MP HA. To determine the highest pH at which the HA proteins promote membrane fusion, syncytia assays were performed in BHK-21 cells. Consistent with the flow cytometry results, MP HA triggered membrane fusion at pH 5.3 when co-expressed with its MP NA partner, and HP HA triggered fusion at pH 5.7 when co-expressed with its HP NA partner (Figure 1F). The syncytia assays were repeated using BHK-21 cells infected with the MP 437-10 and HP YU562 viruses. In virus-infected cells, MP HA was activated to cause membrane fusion at pH 5.2, and HP HA promoted membrane fusion at pH 5.6. Therefore, during either infection or transient expression, HP HA was destabilized by 0.4 pH units compared to MP HA.

**Figure 2:**
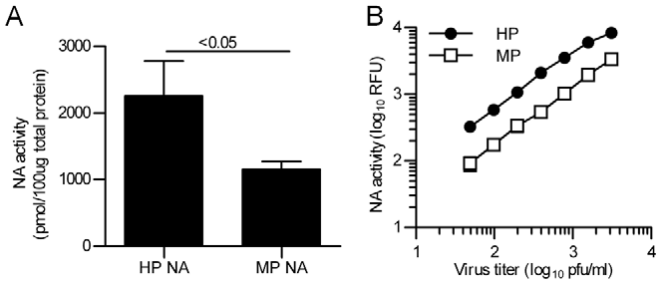
Enzymatic activities of the NA proteins from MP and HP influenza viruses. NA enzymatic activity was determined by using a fluorescence-based assay for transiently expressed NA protein (**A**) and for prestandardized virus (**B**).

We have recently shown that NA enzymatic activity increases the activation pH of the H5N1 HA protein [16]. Therefore, we next investigated whether potential differences in the activities of NA proteins from the HP YU562 and MP 437-10 viruses might affect HA acid stability. We first measured the activities of HP and MP NA when transiently co-expressed with their homotypic HA protein and found that HP NA had significantly more activity than MP NA (*P*<0.05; unpaired two-tailed *t* test) (Figure 2A). Second, we measured the NA activities of HP and MP viruses *in vitro* and found once again that the HP virus had significantly more NA activity than the MP virus (*P*<0.01; unpaired two-tailed *t* test) (Figure 2B). Third, we measured the pH of activation of the HAs when co-expressed with either the HP or MP NA protein (Figure 1F). MP HA was activated at a higher pH of 5.45 when co-expressed with the more active, heterotypic HP NA compared to co-expression with the less active, homotypic MP NA (pH of 5.3). HP HA was activated at a lower pH of 5.4 when co-expressed with the less active, heterotypic MP NA compared to co-expression with the more active, homotypic HP NA (pH of 5.7). In summary, we found that the HA protein from the HP YU562 virus was activated at a higher pH than the HA protein from the MP 437-10 virus, and increased NA activity from the HP YU562 virus was associated with a further increase in the pH of HA activation.

### Residues that regulate HA acid stability

**Figure 3:**
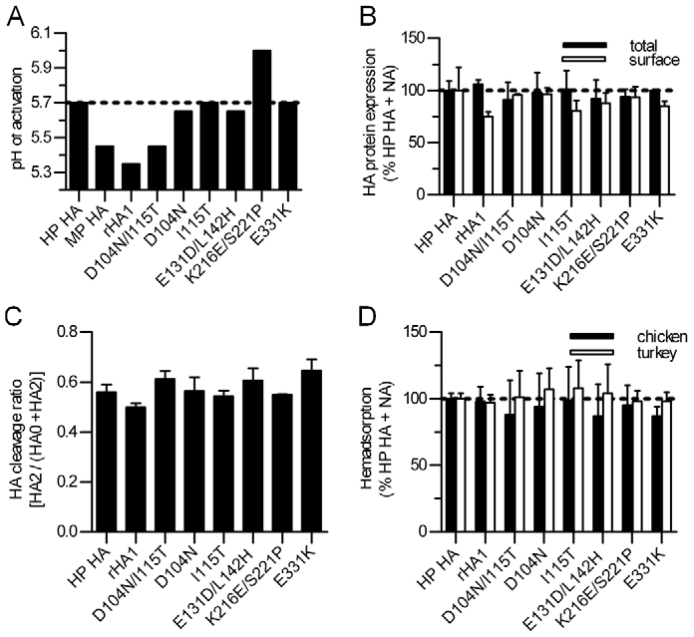
Biochemical properties of wild-type and mutant HA proteins. (**A**) The pH of HA activation was determined as the average of the highest pH at which syncytia formation occurs and the midpoint of the pH of conformational changes. (**B**) HA protein expression. Closed bars represent total expression as determined by using Western blot analysis, and open bars represent cell-surface expression analyzed by flow cytometry. (**C**) HA protein cleavage ratio, measured as described in Figure 1A. (**D**) Hemadsorption of chicken and turkey erythrocytes to cell surface-expressed HA protein. Wild-type and mutant HP HAs were co-expressed in presence HP NA in all experiments. Values depict the average ± standard deviation of at least 3 independent experiments (for total expression and cleavage) or triplicate experiments for surface expression and hemadsorption. HP, highly pathogenic. MP, moderately pathogenic. rHA1, HP HA possessing D104N, I115T, E131D, and L142H mutations.

The HA proteins of MP 437-10 and HP YU562 viruses differ by 7 amino-acid residues in HA1. Residues 104 and 115 are at the ends of the 110-helix in the vestigial esterase sub-domain of the RBD, 131 and 142 are distal to the receptor-binding pocket in the receptor-binding sub-domain, 216 and 221 are at the interface of receptor-binding sub-domain protomers, and 331 is within the polybasic cleavage site [17]. To identify the residues that are responsible for altering HA acid stability, we introduced mutations into the HP HA that correspond to those found in the MP HA, either individually or in combination. We introduced the mutations into the HP HA, rather than vice versa, because chicken LD_50_ values for influenza viruses containing analogous mutations were previously made in the background of the 8 gene segments of the HP virus [17]. We then measured the biochemical properties of the mutant HP HA proteins when co-expressed with the homotypic HP NA protein. We initially focused on three chimeric HP HA proteins: rHA1 (D104N/I115T/E131D/L142H), rHA3 (K216E/S221P), and rHA5 (E331K). None of the three chimeric proteins displayed altered levels of expression, cleavage, or receptor-binding avidity of the HP HA protein (Figure 3). The E331K mutation in the polybasic cleavage site of rHA5 did not affect the HA activation pH (Figure 3A). The mutant HP HA protein containing K216E/S221P mutations (rHA3) was also not responsible for decreasing the pH of activation, but instead had the opposite effect by raising the pH of activation of the HP HA protein to 6.0 (Figure 3A) due to the presence of the K216E mutation (Figure S2).

The rHA1 chimeric HP HA protein containing 4 mutations (D104N/I115T/E131D/L142H) was previously shown to reduce the pathogenicity of HP YU562 virus in chickens [17], and in this study we found that the rHA1 mutations reduced the activation pH of HP HA from 5.7 to 5.35 (Figure 3A), a value similar to that of MP HA. To identify the responsible residue(s), we generated HP HA proteins that contained either single (D104N, I115T, E131D, or L142H) or double (D104N/I115T or E131D/L142H) mutations. A reduced activation pH was only present in the D104N/I115T double mutant, which was triggered at pH 5.45, a value identical to that of the MP HA protein when co-expressed with the HP NA protein (Figure 3). Therefore, our data suggest that changes at residues 104 and 115 contribute to the difference in the activation pH values of the HP and MP HA proteins.

### Relationship between HA acid stability and H5N1 pathogenicity

**Figure 4:**
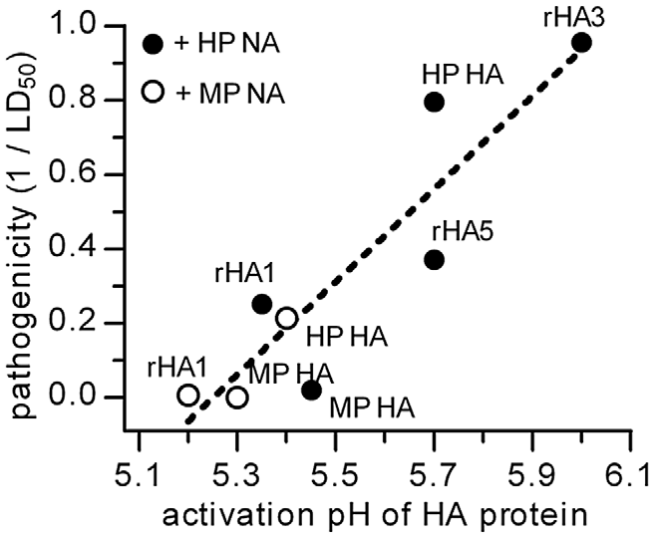
HA activation pH and pathogenicity in chickens. The activation pH values are averages of the midpoint pH of conformational changes and the highest pH at which syncytia form. Pathogenicity is expressed as the reciprocal of the LD_50_ values in chickens.

We next compared our measurements of HA activation pH to the values of 50% lethal dose (LD_50_) in chickens that had been infected with H5N1 viruses containing equivalent HA mutations [17]. An increase in the pH of activation of the HA protein correlated (*R* ^2^ = 0.82) with an increase in pathogenicity, represented as the reciprocal of LD_50_ (Figure 4). HP HA and rHA3 (K216E/S221P) had relatively high activation pH values of 5.7 and 6.0, respectively, and promoted the most pathogenicity in chickens with the lowest LD_50_ values of 0.1 and 0.02 log_10_ EID_50_/mL, respectively. All of the HAs that had activation pH values less than 5.5 promoted reduced pathogenicity in chickens, resulting in LD_50_ values greater than 0.5 log_10_ EID_50_/mL. For HP HA, the presence of the more-active HP NA resulted in an activation pH of 5.7 and an LD_50_ value of 0.1 EID_50_/mL, whereas the presence of the less-active MP NA decreased the activation pH to 5.4 and lowered the pathogenicity to an LD_50_ value of 0.67 EID_50_/mL. Conversely for MP HA, the presence of the less-active MP NA resulted in an activation pH of 5.3 and an LD_50_ value of 3.7 EID_50_/mL, whereas the presence of the more-active HP NA increased the activation pH to 5.45 and enhanced the pathogenicity to an LD_50_ value of 1.7 EID_50_/mL. In some cases when switching NA partners, the relationship between HA activation pH and pathogenicity was less pronounced. For example, the HP HA + MP NA combination resulted in an HA activation pH of 5.4 and an LD_50_ value of 0.67 EID_50_/mL while the MP HA + HP NA combination had a slightly higher activation pH of 5.45 but was less pathogenic with an LD_50_ value of 1.7 EID_50_/mL. While a loss of balance between HA's receptor-binding activity and NA's enzymatic activity [18] could potentially cause such a discrepancy when the NA partner is switched, another unidentified mechanism may play a role instead as the presence of either NA resulted in similar receptor-binding avidities for MP and HP HA (Figure 1C). Overall, though, the data reveal a trend in which an increase in the pH of activation of the HA protein is associated with increased pathogenicity in chickens within the observed pH range of 5.2 to 6.0.

### Crystal structures of H5N1 HA proteins

**Figure 5:**
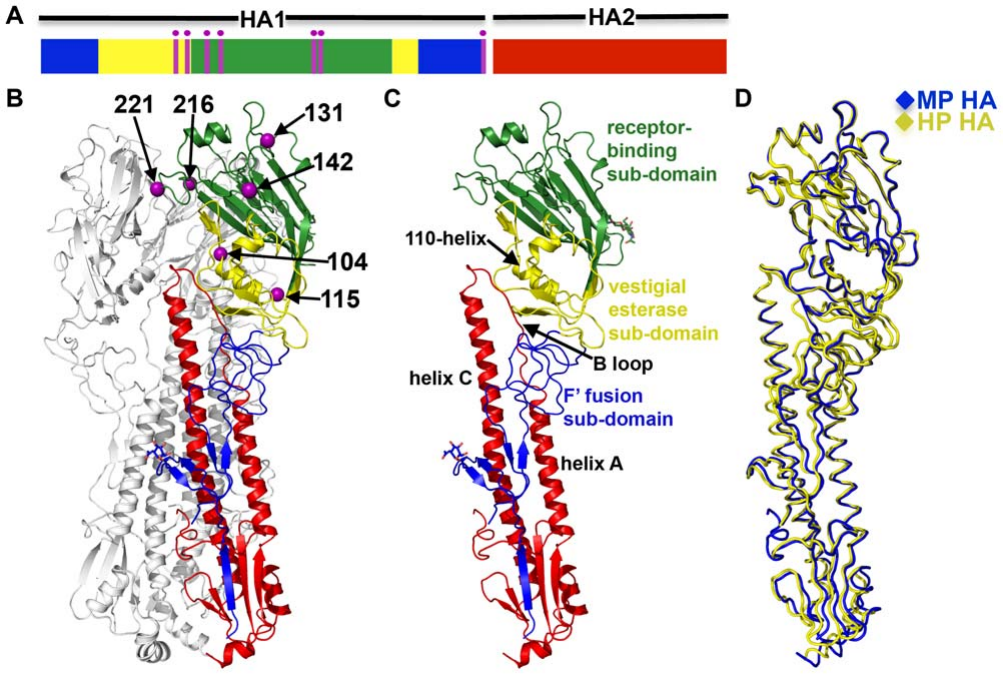
Crystal structures of the MP and HP HA proteins. (**A**) Schematic of the HA protein after proteolytic cleavage into HA1 and HA2 subunits. The receptor-binding domain (RBD) consists of the receptor-binding sub-domain (green) and the vestigial esterase sub-domain (yellow). The F' fusion sub-domain in HA1 is colored blue and the HA2 stalk domain is colored red. The locations of the 7 amino acids that differ between the MP and HP HA proteins are highlighted in magenta. (**B**) Crystal structure of MP HA trimer determined at 2.50Å. One protomer is colored as in panel A (with HA1 in blue, HA2 in red, and 6 amino acids that differ in HP HA shown in magenta spheres). The seventh amino acid (337) that differs in HP HA is removed after cleavage at the polybasic cleavage site between HA1 and HA2. Glycosylation carbohydrates observed in the electron-density maps at HA1 residues Asn34 and Asn169 are shown as a ball-and-stick model. The remaining 2 HA protomers are colored grey. (**C**) Crystal structure of one protomer of MP HA. The domains of the protein are identified with color: receptor-binding sub-domain (green), vestigial esterase sub-domain (yellow), F' fusion sub-domain (blue), and HA2 stalk domain (red). Important structural features include the 110-helix in the vestigial esterase sub-domain and helix A, B loop, and helix C in the HA2 stalk domain. (D) Superposition of 1 protomer from the crystal structure of MP HA (blue) and 2 crystal structures of HP HA (yellow). HP HA crystallized in 2 forms; their structures were determined at 3.10Å (Crystal form 1) and 2.95Å (Crystal form 2). The coordinates for all 3 crystal structures have been deposited at the Protein Data Bank (PDB entry 3S11 [MP HA], PBD entry 3S12 [HP HA Crystal form 1], and PDB entry 3S13 [HP HA Crystal form 2]). All residues are labeled using H3 numbering.

To gain insights into the structural basis for altered HA acid stability, we determined the crystal structures of the prefusion forms of the MP and HP HA proteins (Table 1). One crystal form of the MP HA protein was obtained at pH 8.5, and two crystal forms of the HP HA protein were obtained at pH 6.6, all above the low pH thresholds for conformational changes for the two proteins. The overall folds of the MP and HP HA proteins (H5N1 clade 3) are very similar to each other (Figures 5D and S3) and to those of the previously determined HA structures from isolates A/Vietnam/1203/04 and A/Vietnam/1194/04 (H5N1 clade 1) [22], [23]. Of the seven differing residues, 331 is in the polybasic cleavage site and is not present in the cleaved structures, 104 and 115 are within the vestigial esterase sub-domain in the RBD, and 131, 142, 216, and 221 are within the receptor-binding sub-domain in the RBD (Figure 5B,C).

**Table 1:** Crystallographic data.

Data collection*			
Crystal	HP HACrystal form 1	HP HA Crystal form 2	MP HA
Space group	P321	P321	P2_1_
*a*, *b*, *c* (Å)	112.6, 112.6, 134.7	111.9, 111.9, 192.1	69.4, 241.1, 70.1
α, β, γ (°)	90.0, 90.0, 120.0	90.0, 90.0, 120.0	90.0, 116.7, 90.0
Resolution (Å)	50.0–3.1 (3.21–3.10)	50.0–2.95 (3.06–2.95)	50.0–2.5 (2.59–2.50)
R_merge_	0.151 (0.459)	0.119 (0.480)	0.104 (0.431)
*I*/*σI*	17.7 (5.3)	22.8 (6.1)	17.7 (2.3)
Completeness (%)	100 (99.9)	99.9 (99.7)	96.0 (72.8)
Redundancy	11.0 (9.8)	12.6 (11.1)	4.8 (2.8)
Refinement			
Resolution (Å)	50.0–3.1	50.0–2.95	50.0–2.5
No. reflections	17,391	28,027	64,349
*R*_work_/*R*_free_ †	0.217 / 0.264	0.238 / 0.251	0.221 / 0.265
Ramachandran (%)			
Favored	92.1	92.4	95.1
Allowed	7.9	7.6	4.9
Outliers	0.0	0.0	0.0
RMS deviations			
Bond lengths (Å)	0.008	0.009	0.007
Bond angles (°)	1.117	1.287	1.020

*Data were collected from a single crystal. Values of the highest-resolution shell are shown in parentheses.

†*R*_free_ was calculated using 5% of the reflections.

An overlay of the RBD, which includes the receptor-binding sub-domain and the vestigial esterase sub-domain, shows that the six varying residues induce no substantial differences in the α-carbon backbone structures of two prefusion HA strains (Figure 5D). The RMSD values (on α-carbons) between the RBDs of MP HA and HP HA crystal forms 1 and 2 are 0.31 Å and 0.27 Å, respectively. The receptor-binding pocket residues had no differences in conformation between the MP and HP HA structures, and mutations at residues 131 and 142 distal to the receptor-binding pocket did not alter the backbone structure (Figure S4). The most significant differences occur between the two crystal forms of the HP HA protein, specifically, in the B loops of the HA2 stalk domain along with parts of the vestigial esterase sub-domain (not including the 110-helix) and the F' fusion sub-domain (Figures 5C and S5A-C). Both crystal forms were grown in identical solutions at pH 6.6, 0.9 pH units above its activation pH of 5.7, but display different crystal-packing interactions in the B loop region. The HA2 B loops from H5N1 isolates VN1194 and VN1203 also adopt “in” and “out” forms, respectively (Figure S5F), despite both proteins having identical residues in this region and their crystals being grown at similar pH values of 6.5 and 6.55 [22], [23]. VN1203, which adopts the “out” form in the absence of antibody, adopts the “in” form when bound to antibody [24], [25] (Figure S5F). Because the various prefusion H5N1 HA proteins adopt either “in” or “out” forms of the B loop in similar conditions and with little apparent structural consequence, the apparently flexible B loop may have little functional relevance in the prefusion conformation of H5N1 HA.

**Figure 6:**
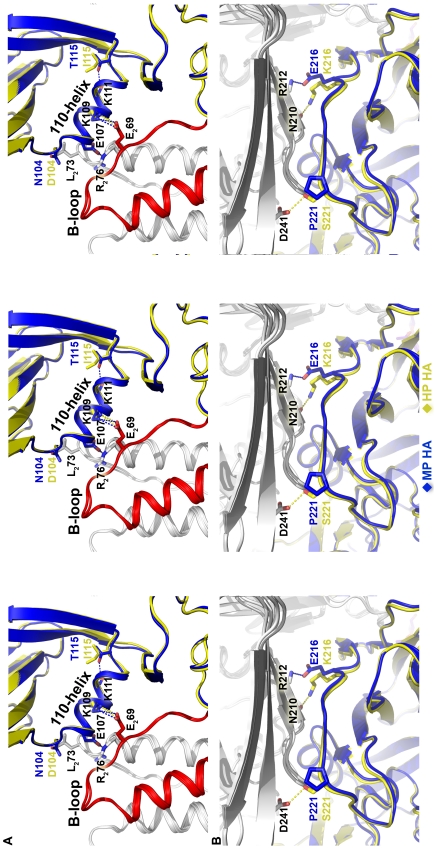
Structures at sites of sequence variation between the MP and HP HA proteins. (**A**) Zoomed-in stereo view of residues 104 and 115 at the N- and C-terminal ends of the 110-helix of HA1 in MP HA (blue) and HP HA (yellow). The corresponding HA2 backbone is colored red and HA2 sidechains of the other two protomers are colored white. Residues from HA2 are denoted with a “2” subscript. Dotted lines represent hydrogen bonds and are colored to match the corresponding HA protein with MP HA in blue and HP HA in yellow. Note the interaction between the 110-helix with the B loops from 2 HA2 protomers. (**B**) Zoomed-in stereo view of residues 216 and 221 in MP HA (blue) and HP HA (yellow) and their location at the trimer interface. The adjacent protomers across the RBD-RBD interface for MP and HP HA are all shown in gray. In both **A** and **B**, the left and middle panels represent the divergent pair of stereoimages while the middle and right panels represent the convergent pair of stereoimages. All residues are labeled using H3 numbering.

Residues 216 and 221 in the receptor-binding sub-domain interact with residues in adjacent monomers across the RBD trimer interface (Figure 5B). In MP HA, E216 makes a hydrogen bond with neighboring RBD backbone amide R212 (Figure 6B), although this hydrogen bond is found only in two of the three E216 residues in the HA trimer. The lack of hydrogen bonding by the third E216 could be biologically relevant, helping to destabilize HA, or could be due to a crystallographic artifact. In HP HA, K216 makes a hydrogen bond with neighboring RBD backbone carbonyl N210 (Figure 6B). We found that a K216E mutation increased the pH of activation of HP HA by 0.4 pH units (Figure S2). However, the presence of an E216 residue did not seem to have a dominant effect in the context of MP HA, which contains six other mutations and has an overall decreased pH of fusion compared to HP HA (Figure 1). Compared to P221 in MP HA, S221 in HP HA forms a hydrogen bond to the D241 side-chain in an adjacent monomer across the RBD-RBD interface (Figure 6B). While one might have expected that a S221P mutation would destabilize HA by breaking a hydrogen bond and introducing a proline, we found that an S221P mutation had the opposite effect in the background of HP HA, decreasing the activation pH from 5.7 to 5.5 (Figure S2A). Perhaps the rigidity of a proline at residue 221 stabilizes this region. It has been previously hypothesized for H5N1 and demonstrated for H3N2 (X31 strain) that HA stability is regulated by salt bridges across the RBD-RBD interface and that an alteration in HA stability may play a role in influenza virus infectivity [26]. In the present study, the structural and biochemical results on H5N1 mutations at residues 216 and 221 show that alterations in hydrogen-bonding interactions across the RBD-RBD interface also regulate H5N1 HA acid stability (Figure 6B, S2).

The higher activation pH of HP HA compared to MP HA was largely mapped to differences in residues 104 and 115 (Figure 3A), which are located at the N- and C-termini of the 110-helix in the vestigial esterase sub-domain of the RBD (Figure 5). In the prefusion conformation, the 110-helix in HA1 interacts with the interhelical B loops of two protomers in HA2 (Figure 6A). Most notably, HA1 residues E107 and K109 in the 110-helix form salt bridges with HA2 residues R76 and E69, respectively. This interaction may stabilize the metastable structure by preventing the B loops from springing out to their coiled-coil form or, alternatively, by stabilizing the RBD head domains to prevent their dissociation from the HA2 stalk after B loop structural changes are initiated by low pH [27]. These salt bridges are clearly important because E107 and K109 are more than 99.7% conserved amongst sequenced H5N1 HA proteins, and R76 and E69 are 100% conserved. The structures suggest that the sequence variations at residues 104 and 115 modulate the stability of the 110-helix and, consequently, the interactions between the 110-helix and the stalk domain. We hypothesize that the negatively charged D104 in HP HA forms less favorable interactions with the neighboring L73 at the top of the HA2 coil in the prefusion conformation than does the polar N104 in MP HA (Figure 6A). T115 in MP HA forms a hydrogen bond with the backbone carbonyl oxygen of L111, thereby capping and stabilizing the C-terminal end of the 110-helix. I115 in the HP HA does not form this hydrogen bond, and the stabilizing capping interaction is therefore absent. Overall, we suggest that the combination of D104N and I115T mutations found in MP HA promotes the stabilization of the 110-helix and thereby stabilizes the prefusion form of the HA protein, lowering its pH of activation and, consequently, attenuating the virus.

## Discussion

The goal of the current study was to understand how amino acid variations in the HA protein contribute to differences in pathogenicity between two H5N1 influenza virus isolates. Our analyses revealed that H5N1 pathogenicity in chickens correlates with the activation pH of the HA protein. Specifically, an increase in the pH of activation of the HA protein from 5.3 to 5.7 was associated with the greater pathogenicity of the A/chicken/Hong Kong/YU562/2001 isolate in chickens compared to the A/goose/Hong Kong/437-10/1999 isolate. Other factors are largely similar including their prefusion structures, expression levels, cleavage levels, and receptor-binding properties. We have also shown that naturally occurring mutations in the HA proteins of circulating H5N1 influenza viruses have altered the acid stability of the HA protein. Six of the 7 available HA protein sequences from H5N1 viruses sampled in 1999 (Table 2), including the MP 437-10 isolate, contain the N104 and T115 residues that we found to contribute to a reduced HA activation pH and reduced pathogenicity. In contrast, none of the available 2847 HA protein sequences obtained since 2000 contain the N104/T115 combination, whereas approximately 93% contain the D104/I115 combination found in the HP YU562 isolate that leads to an increased HA activation pH and increased pathogenicity. These epidemiological observations suggest that there has been a negative selection pressure against the N104/T115 combination of residues that are found in the MP virus that has prevented its propagation in avian species, and this may be related to the relatively low pH that is required to trigger membrane fusion.

**Table 2:** Prevalance of amino-acid residues at positions 104 and 115 of the HA protein.

Amino acid residue		Prevalance
104	115	(%, n = 2938*)
N	T	0.20
N	I	4.08
N	V	0.20
D	T	0.24
D	I	93.47
D	F, L, or V	1.39
A, E, G, S, V, or Y	I	0.47

*HA gene sequences of H5N1 viruses collected between 1996 and May 2011 are included. Boldface and underlined type indicate the amino acid combinations at positions 104 and 115 in the MP and HP HA proteins, respectively.

Here, we found that sequence variations in the RBD (which includes the receptor-binding and vestigial esterase sub-domains) do not alter the structure of the prefusion RBD but instead modulate the activation pH of the H5N1 HA protein. While such a phenotype may be unexpected, a D112G mutation in the HA2 stalk domain of A/Aichi/68 (H3N2) has also been shown to alter HA acid stability yet involves only the replacement of the Asp sidechain with a water molecule at the mutation site, causing no detectable changes in the backbone or surrounding protein structure in prefusion HA [28]. Further evidence that the RBD forms a stable structure in the prefusion conformation is suggested by the fact that the isolated RBD from A/H1N1/2009, *E. coli*-expressed and refolded [29], has recently been shown to adopt the same fold [30] as the RBD in the intact, prefusion HA ectodomain [31], [32]. The structures of a mutant H2N2 HA protein (A/Japan/305/57) determined from crystals grown at pH 8.1 and 5.3 suggest that early structural changes in HA after acid activation include bulging out of the HA2 B loop and distortions in the HA1 vestigial esterase and F' fusion sub-domains (Figure S5G-I) [27]. These reversible structural changes were suggested to correspond to an early intermediate of the HA protein after acid activation and may help initiate global HA refolding. We also observed the two forms of the B loop, vestigial esterase sub-domain, and F' fusion sub-domain in the two different crystal forms of the HP A/chicken/Hong Kong/YU562/2001 (H5N1) HA protein (Figure S5A-C), although both H5N1 HA crystals were grown in identical solutions at pH 6.6 (0.9 pH units above its pH of activation). While the two HP H5N1 HA structures suggest that the observed differences in the B loops, vestigial esterase sub-domain, and F' fusion sub-domain in the prefusion structures reported here are due to differing crystallographic environments, it is possible that upon low-pH activation the H5N1 HA protein favors the “out” form of the B loop and pivoting of the F' fusion sub-domain similar to that which is observed when the H2 HA protein is exposed to acidic pH [27].

Mutations to amino-acid residues other than 104 and 115 can modulate HA protein activation and influenza virus pathogenicity. For example, in a proof-of-concept study, we recently showed that mutations to conserved residues in the stalk domain (albeit, mutations that have not been observed in circulating H5N1 viruses) alter HA acid stability and, as a result, modulate H5N1 replication, pathogenicity, and transmissibility in ducks [16]. In that study, recombinant A/chicken/Vietnam/C58/2004 (H5N1, clade 1) viruses containing HA proteins activated at pH values of 5.6 and 5.9 were highly virulent and transmissible in mallards, while those activated at pH values of 5.4 and 6.3 were avirulent and not transmissible. Taken together, our previous [16] and present data suggest that high levels of H5N1 influenza virus infection and pathogenicity in avian species may be supported by a relatively narrow range of HA protein activation pH values, minimally pH 5.6 to 6.0.

In general, opposing pressures may limit the activation pH of the HA protein to an optimal range that may shift depending on viral and host factors. A relatively low pH of HA protein activation would be needed to avoid inactivation in the environment or in mildly acidic tissues, whereas the activation pH would still need to be high enough to allow membrane fusion to occur before the virus is trafficked to the lysosome. Circumstantial and direct evidence support this notion. First, the acid stabilities of influenza virus HA proteins range from pH 4.6 to 6.0 and vary by subtype and host species [33]. Second, the adaptation of H3N2 viruses from eggs to mammalian cells [15] and of H7N3 viruses from ducks to turkeys [14] resulted in HA mutations that altered the acid stability of the HA protein. Third, in the presence of high concentrations of amantadine, a compound that raises endosomal pH, resistant variants of H3N2, H7N1, and H7N7 viruses have been selected that have increased HA activation pH values [34]–[37].

In the present work, a higher level of NA enzymatic activity contributed to an increase in the activation pH of the HA protein and was associated with greater virulence by HP YU562 virus compared to MP 437–10 virus. Compared to expression of HA alone, coexpression of NA together with HA has previously been shown to increase the pH of activation of the H5N1 HA protein by 0.5 pH units in the absence but not the presence of the NA inhibitor oseltamivir [16], further demonstrating a link between NA activity and destabilization of the HA protein. The mechanism by which NA enzymatic activity augments HA protein fusogenic activity is unknown; however, cleavage of sialic-acid containing N-linked glycosylation sites on the HA protein may decrease the energy required to trigger HA conformational changes by destabilizing the prefusion form of individual HA trimers. Alternatively, enhanced cleavage of HA glycosylation sites could potentially promote the synchronized activation and refolding of adjacent, interacting HA trimers [38]. Increased NA enzymatic activity could also reduce interference that would occur if HA trimers bound to other HA trimers, NA proteins, glycoproteins, or glycolipids. However, a very large reduction in NA enzymatic activity might be needed to cause such interference in the first place. The importance of glycosylation sites in regulating HA activation and influenza virus replication has been demonstrated previously for A/FPV/Rostock/34 (H7N1), whose HA protein is destabilized by the removal of glycosylation sites in the stalk domain [39]. Complementation of HA protein fusogenic activity by NA enzymatic activity may depend on influenza virus subtype. For example, NA co-expression resulted in increased membrane fusion by the HA proteins from HPAI H7N4 and human H1N1 influenza viruses [20], while the addition of exogenous neuraminidase had no effect on membrane fusion mediated by human H2N2 and H3N2 HA proteins but instead led to an increase in receptor-binding activity by HA [40], [41].

Influenza virus pathogenicity is a polygenic trait that is modulated by a combination of viral and host factors [1], [6], [7]. Although an optimal activation pH of the HA protein appears to be necessary for high pathogenicity by H5N1 influenza viruses in avian species, we do not expect it to be sufficient to promote high pathogenicity in the absence of a polybasic cleavage site, host-appropriate receptor-binding specificity, or an efficient polymerase. Moreover, if the optimal activation pH differs between avian and mammalian species, additional studies will be needed to determine whether alterations in HA acid stability may contribute to the pandemic potential of H5N1 influenza viruses.

## Materials and Methods

### Cloning

HA genes from A/goose/Hong Kong/437-10/1999 (MP) and A/chicken/Hong Kong/YU562/2001 (HP) H5N1 influenza viruses were cloned into pHW2000, pCAGGS, and pAcGP67B plasmids as described previously [16], [19], [22]. Point mutations were introduced by QuickChange mutagenesis (Stratagene).

### Transient expression of HA and NA

Monolayers of Vero or BHK-21 cells at 70% to 80% confluency in 6-well plates were transiently transfected with pCAGGS HA (1.0 µg) and pCAGGS NA (0.1 µg) plasmids by using a Lipofectamine Plus expression system (Invitrogen) [19]. After 4 hours at 37°C, the transfection medium was replaced with DMEM containing 10% fetal bovine serum (and 1% glutamine for BHK-21 cells), and cells were incubated for 16 h at 37°C.

### Expression and activation pH of HA protein

Biochemical analyses were performed as described previously [19]. Briefly, HA proteins were resolved on 4-12% NuPAGE BisTris polyacrylamide-SDS gels (Invitrogen) and visualized on a Typhoon 9200 imager (GE Healthcare, Waukesha, WI). HA surface expression was determined by using flow cytometry with the primary monoclonal HA antibody Vn04-02 (1∶2000) and fluorescein-conjugated, AffiniPure donkey anti-mouse IgG (H+L, Jackson Immuno Research, West Grove, PA) secondary antibody. The pH of HA conformational changes (to 0.1 pH resolution) was determined by using flow cytometry and monoclonal antibodies Vn04-09 and Vn04-16, which preferentially bind to the prefusion and postfusion HA forms, respectively [21]. To determine the pH of membrane fusion, BHK-21 cell monolayers were transfected with pCAGGS HA and pCAGGS NA plasmids as described above or infected with viruses at an MOI of 3 PFU/cell. At 16 h posttransfection or 6 h postinfection, cells were washed and overlaid with PBS+ (PBS containing calcium and magnesium at 0.1 g/liter) with the pH adjusted to 0.1 resolution with 0.1 M citric acid. The pH of fusion was expressed as the highest pH at which syncytium formation was observed.

### Hemadsorption

Sixteen hours after transfection, monolayers of Vero cells were washed twice with PBS+, overlaid with 1% chicken or turkey erythrocytes, and incubated at 37°C for 30 min. Monolayers were then washed 3 times with DMEM (phenol red-free) to remove unbound red blood cells and lysed with 1X RBC lysis buffer (eBioscience). The amount of bound erythrocytes was determined by measuring the absorbance of clarified lysate at 415 nm by using a Synergy-2 Multi-mode microplate reader (BioTek, Winooski, VT).

### NA enzymatic activity

NA enzymatic activity was determined by using a fluorescence-based NA assay with methyl umbelliferone N-acetyl neuraminic acid (MUNANA; Sigma, St Louis, MO) as a substrate (final concentration of 100 µM) [42]. Fluorescence due to release of 4-methylumbelliferone was measured by using a Synergy-2 Multi-mode microplate reader. The enzyme activity of transiently expressed NA was determined as the quantity (pmol) of 4-methylumbelliferone sodium salt (Sigma) generated during a 30 min incubation at 37°C and was standardized to 0.1 mg total protein by using a bicinchoninic acid assay (Sigma). The NA enzyme activity of varying titers of HP and MP viruses was standardized to the relative PFU/mL titers.

### HA ectodomain production

Purified ectodomains of the HP and MP HA proteins were prepared as described previously [22] using a baculovirus expression system (BD Biosciences) and Sf9 insect cells. Secreted HA ectodomains were purified by metal affinity chromatography followed by thrombin digestion of the purification tag. Trypsin was used to cleave HA into the active HA1/HA2 form. HA proteins were further purified by size-exclusion chromatography and concentrated to 1.4 mg/mL (MP HA) or 3.0 mg/mL (HP HA).

### Crystal structure determination

HA protein crystals were grown by the hanging-drop vapor diffusion method at 18°C. MP HA crystallized in a well solution of 23% PEG 3350 and 0.1 M Tris-HCl, pH 8.5. From HP HA, two crystal forms were obtained in the same crystallization conditions (1.62 M ammonium sulphate, 0.1 M sodium cacodylate, pH 6.6). Crystals were transferred to a well solution containing 25% glycerol (MP HA) or 25% ethylene glycol (HP HA) for 1–2 minutes before freezing in liquid nitrogen. Diffraction data were collected at cryogenic temperature at X-ray wavelength 1.00 Å from the Southeastern Regional Collaborative Access Team's 22-ID and 22-BM beamlines at the Advanced Photon Source (Argonne National Laboratory, Chicago, IL). Data processing and reduction was completed by using HKL-2000 software [43].

HA ectodomain structures were determined by molecular replacement using the program Phaser [44]. From HP HA crystal form 1, a solution was obtained by using a single HA protomer from the crystal structure of the HA from H5N1 A/Vietnam/1203/2004 (PDB entry 2FK0). For MP HA, the HP HA crystal form 1 structure was used as a molecular replacement model. For HP HA crystal form 2, the best molecular replacement solution was obtained by using a single HA protomer from MP HA's crystal structure. Model building was performed by using Coot [32] followed by iterative rounds of simulated annealing using Phenix [45] and restrained refinement using the CCP4 software suite's REFMAC5 [46]. Refinement was monitored by following the *R*_free_ value calculated for a random subset (5%) of reflections omitted from refinement. The final models were validated by using MolProbity [47] and are numbered according to H3 numbering based on the crystal structure of A/Vietnam/1203/2004 H5 HA (PDB entry 2FK0). We used the H3 numbering scheme in this manuscript, which differs from the H5 numbering that was used previously [17].

After simulated annealing of HP HA crystal form 2, the electron density for the region of the stalk domain that is closest to the viral membrane was very poor due to irregular crystal packing within this region. The structural model of HP HA crystal form 2 was guided by B-factors: residues with B-factors higher than 90 were not included in the model. The final model of HP HA crystal form 2 contains HA1 residues 43–312 and HA2 residues 59–101 (H3 numbering).

### HA protein sequence analysis

HA protein sequences published between 1996 and 2011 (as of May 2011) were obtained from NCBI's Influenza Virus Resource database (http://www.ncbi.nlm.nih.gov/genomes/FLU/). Laboratory sequences or sequences that did not cover the amino-acid positions of interest in this study were excluded. Sequences were aligned by using the ClustalW tool included in BioEdit v7.0.9 [48]. The frequencies of amino-acid residues were calculated for HA1 positions 104, 107, 109, and 115 and HA2 positions 73, 69, and 76 (in H3 numbering).

## Supporting Information

Figure S1 Expression of HA proteins cotransfected with NA proteins in Vero cells. (A) Surface expression of wild-type HP and MP HA proteins cotransfected with wild-type NA proteins as measured by flow cytometry. (B and C) Surface expression of wild-type (wt) and mutant HP HA proteins cotransfected with the HP NA protein as measured by flow cytometry. (D–F) Confocal microscopic images showing expression of HA proteins on unpermeabilized Vero cells. (D) Confocal images of HP HA cotransfected with HP NA. (E) Confocal images of MP HA cotransfected with MP NA. (F) Confocal images of untransfected (mock) cells. At 16 h posttransfection, Vero cells were stained with conformation-independent Vn04-02 mouse monoclonal primary antibody. FITC-conjugated antibody and Alexa Fluor 555-conjugated antibody were used as secondary antibodies for flow cytometry and confocal microscopy, respectively. Untransfected cells were used as a control (mock). For confocal microscopy, nuclear staining was performed using DAPI, 10 µm scale bars are shown, and a Zeiss LSM 510 META laser scanning confocal microscope was used.
(TIF)

Figure S2 Biochemical characterization of mutant HP HA proteins. (A) The pH of HA protein activation determined as the average of the pH values of conformational change and those of syncytia formation. (B) HA protein expression. Closed bars represent total expression as determined by using Western blot analysis, and open bars represent cell-surface expression analyzed by flow cytometry. (C) HA protein cleavage ratio. (D) Hemadsorption of chicken and turkey erythrocytes to cell surface-expressed HA normalized to 100% HP HA hemadsorption. Wild-type and mutant HP HA proteins were co-expressed in the presence of the HP NA protein in all experiments. Values shown are average ± standard deviation of at least 3 independent experiments (for total expression and cleavage) or triplicate experiments (for surface expression and hemadsorption). Asterisks indicate a significant difference (P<0.01) as determined by unpaired two-tailed t-test. HP, highly pathogenic.
(TIF)

Figure S3 Crystal structures of MP HA and HP HA proteins. (A) Crystal structure of MP HA trimer determined at 2.50Å. One protomer is colored with HA1 in blue and HA2 in red. Glycosylation carbohydrates observed in the electron-density maps at HA1 residues Asn34 and Asn169 are shown as a ball-and-stick model. The remaining 2 HA protomers are colored grey. (B) Crystal form 1 structure of HP HA trimer determined at 3.10Å. (C) Crystal form 2 structure of HP HA trimer determined at 2.95Å. Part of the structure is missing because it is packed in a random fashion throughout the crystal.
(TIF)

Figure S4 Zoomed-in stereo view of residues 131 and 142 and their location with respect to the receptor-binding site in MP HA (blue) and HP HA (yellow). Dotted lines represent hydrogen bonds and are colored to match the corresponding HA protein. The left and middle panels represent the divergent pair of stereoimages while the middle and right panels represent the convergent pair of stereoimages. All residues are labeled using H3 numbering.
(TIF)

Figure S5 Comparison of HA structures. (A) Superposition of one protomer from the 2 crystal structures of HP HA. (B) Superposition of the HA1 chains from the 2 crystal structures of HP HA. (C) Superposition of the HA2 chains from the 2 crystal structures of HP HA. The variation between the interhelical B loops (“in” or “out” conformations) in the HP HA structures from two crystal forms at the same pH is likely the result of crystal packing differences. (D) Superposition of 1 protomer from four H5N1 HA crystal structures: VN1194 (PDB entry, 2IBX), VN1203 (PDB entry, 2FK0), VN1203 bound to antibody F10 (PDB entry 3FKU), and VN1203 bound to antibody CR6261 (PDB entry 3GBM). For clarification, the bound antibodies are not shown in the figure. (E) Superposition of the HA1 chains from the four H5N1 crystal structures in D. (F) Superposition of the HA2 chains from the four H5N1 crystal structures in D. (G) Superposition of one protomer from two H2 HA crystal structures. H2 HA (P63) corresponds to PDB entry 3QQB and H2 HA (P21) corresponds to PDB entry 3QQO. (H) Superposition of the HA1 chains from the two crystal structures of H2 HA. (I) Superposition of the HA2 chains from the two crystal structures of H2 HA. The crystallization space groups are described in parentheses; the crystallization pH is also indicated.
(TIF)
