# Supplementary figures and images for: Acid Stability of the Hemagglutinin Protein Regulates H5N1 Influenza Virus Pathogenicity
Source: PLoS Pathog. 2011 Dec 1;7(12):e1002398. doi: 10.1371/journal.ppat.1002398 (PMC3228800; doi:10.1371/journal.ppat.1002398)

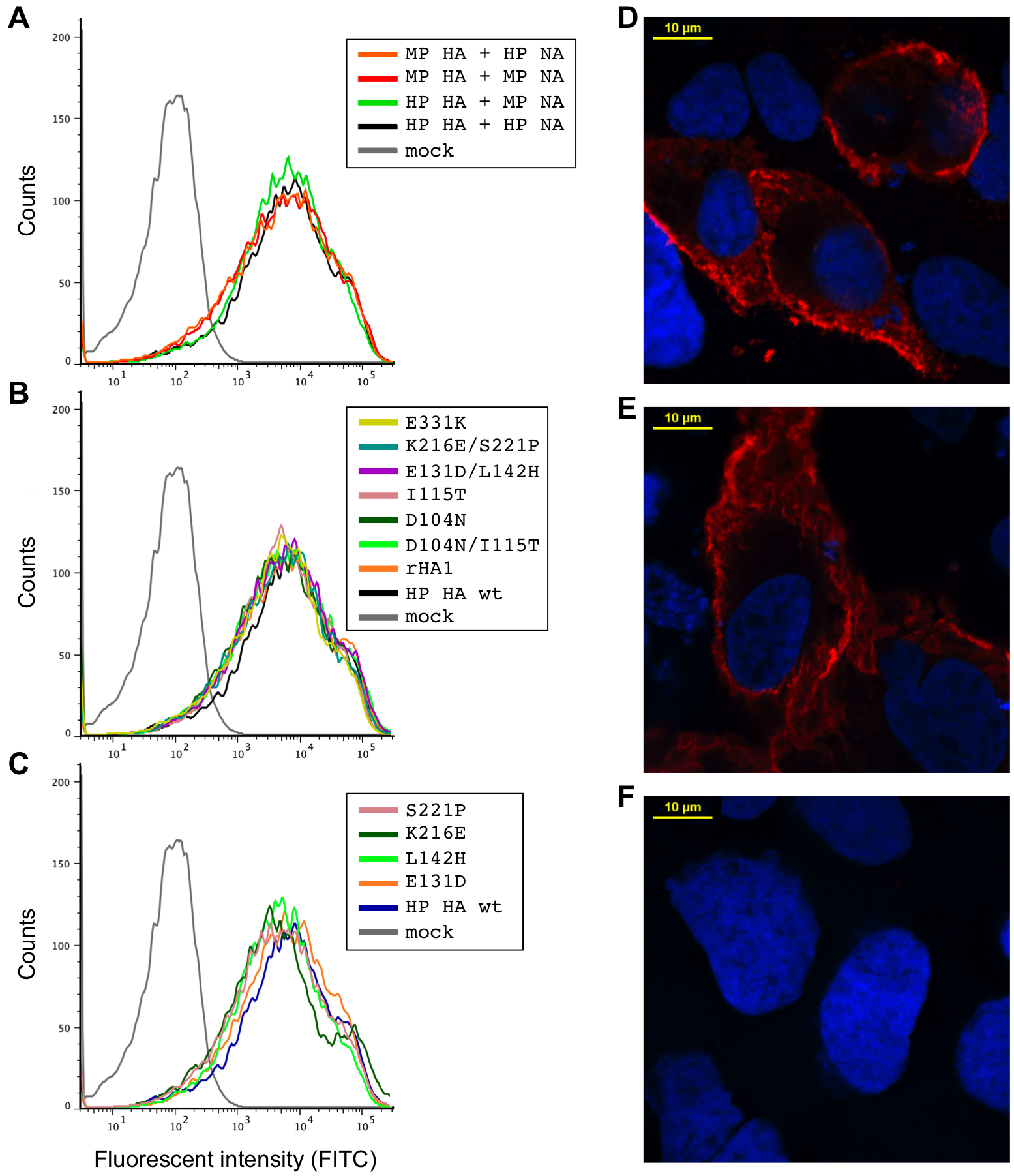

Supplement: Figure S1 — Expression of HA proteins cotransfected with NA proteins in Vero cells. (A) Surface expression of wild-type HP and MP HA proteins cotransfected with wild-type NA proteins as measured by flow cytometry. (B and C) Surface expression of wild-type (wt) and mutant HP HA proteins cotransfected with the HP NA protein as measured by flow cytometry. (D–F) Confocal microscopic images showing expression of HA proteins on unpermeabilized Vero cells. (D) Confocal images of HP HA cotransfected with HP NA. (E) Confocal images of MP HA cotransfected with MP NA. (F) Confocal images of untransfected (mock) cells. At 16 h posttransfection, Vero cells were stained with conformation-independent Vn04-02 mouse monoclonal primary antibody. FITC-conjugated antibody and Alexa Fluor 555-conjugated antibody were used as secondary antibodies for flow cytometry and confocal microscopy, respectively. Untransfected cells were used as a control (mock). For confocal microscopy, nuclear staining was performed using DAPI, 10 µm scale bars are shown, and a Zeiss LSM 510 META laser scanning confocal microscope was used. (TIF) [file ppat.1002398.s001.tif]

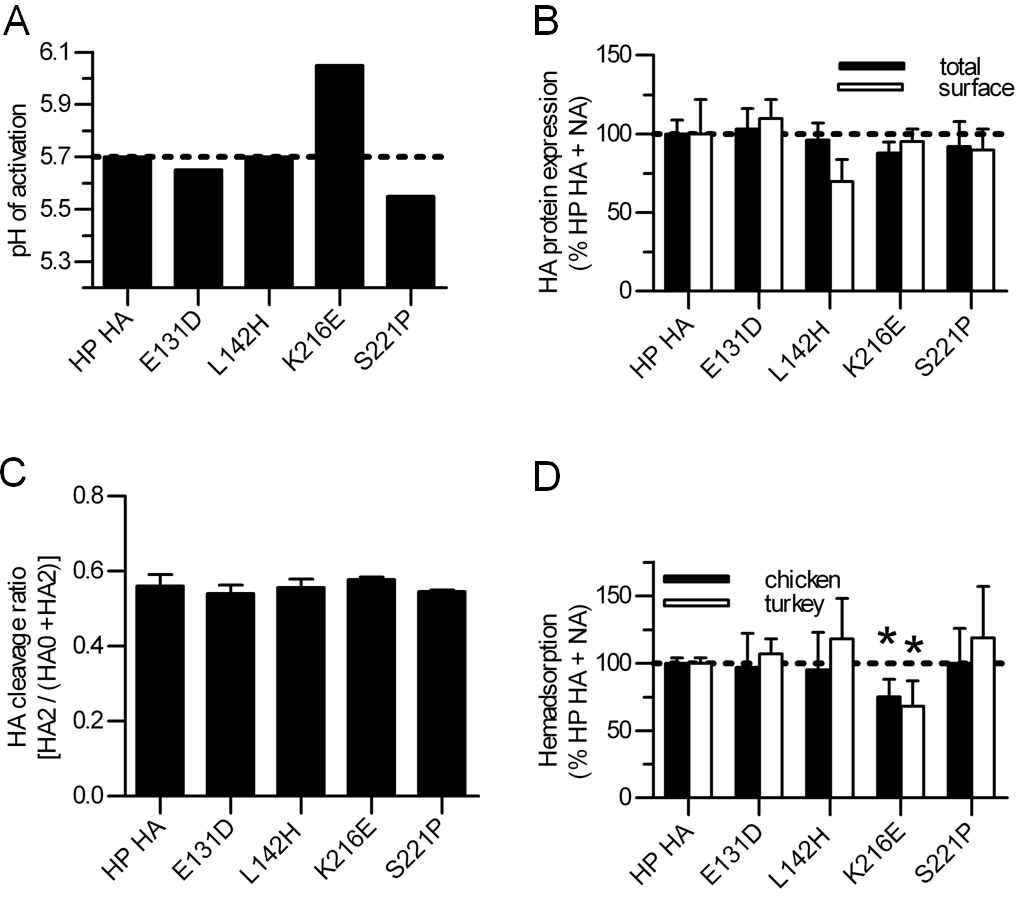

Supplement: Figure S2 — Biochemical characterization of mutant HP HA proteins. (A) The pH of HA protein activation determined as the average of the pH values of conformational change and those of syncytia formation. (B) HA protein expression. Closed bars represent total expression as determined by using Western blot analysis, and open bars represent cell-surface expression analyzed by flow cytometry. (C) HA protein cleavage ratio. (D) Hemadsorption of chicken and turkey erythrocytes to cell surface-expressed HA normalized to 100% HP HA hemadsorption. Wild-type and mutant HP HA proteins were co-expressed in the presence of the HP NA protein in all experiments. Values shown are average ± standard deviation of at least 3 independent experiments (for total expression and cleavage) or triplicate experiments (for surface expression and hemadsorption). Asterisks indicate a significant difference (P<0.01) as determined by unpaired two-tailed t-test. HP, highly pathogenic. (TIF) [file ppat.1002398.s002.tif]

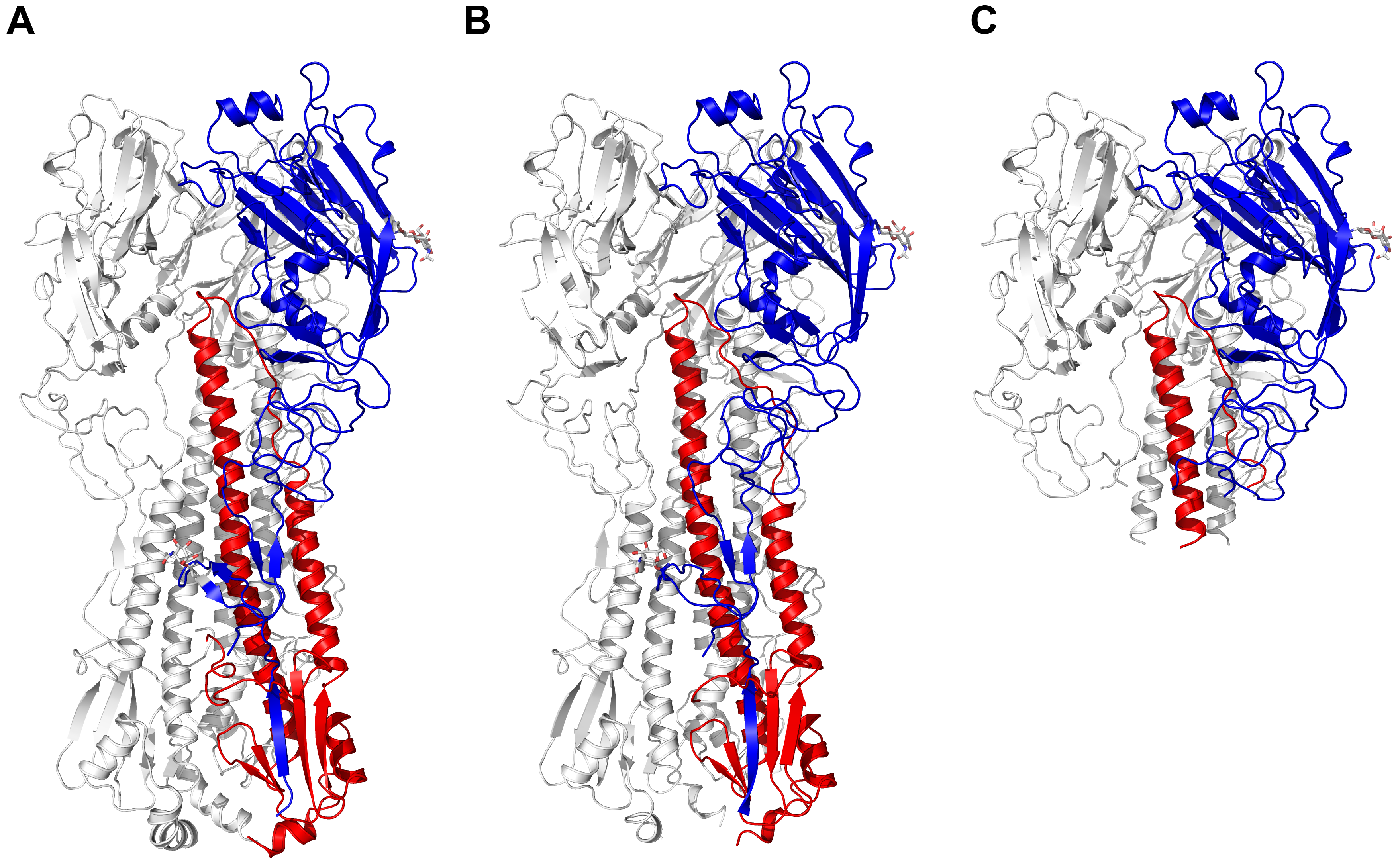

Supplement: Figure S3 — Crystal structures of MP HA and HP HA proteins. (A) Crystal structure of MP HA trimer determined at 2.50Å. One protomer is colored with HA1 in blue and HA2 in red. Glycosylation carbohydrates observed in the electron-density maps at HA1 residues Asn34 and Asn169 are shown as a ball-and-stick model. The remaining 2 HA protomers are colored grey. (B) Crystal form 1 structure of HP HA trimer determined at 3.10Å. (C) Crystal form 2 structure of HP HA trimer determined at 2.95Å. Part of the structure is missing because it is packed in a random fashion throughout the crystal. (TIF) [file ppat.1002398.s003.tif]

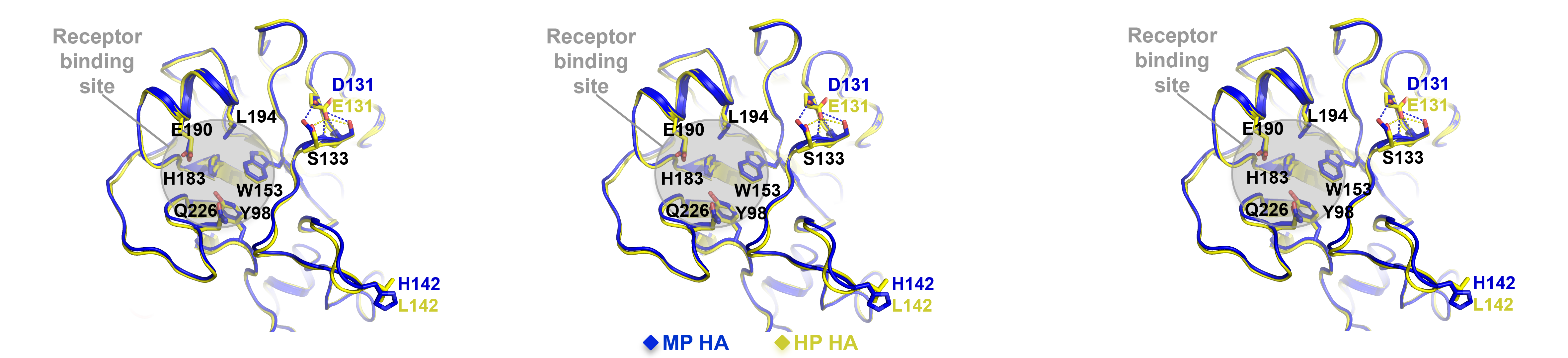

Supplement: Figure S4 — Zoomed-in stereo view of residues 131 and 142 and their location with respect to the receptor-binding site in MP HA (blue) and HP HA (yellow). Dotted lines represent hydrogen bonds and are colored to match the corresponding HA protein. The left and middle panels represent the divergent pair of stereoimages while the middle and right panels represent the convergent pair of stereoimages. All residues are labeled using H3 numbering. (TIF) [file ppat.1002398.s004.tif]

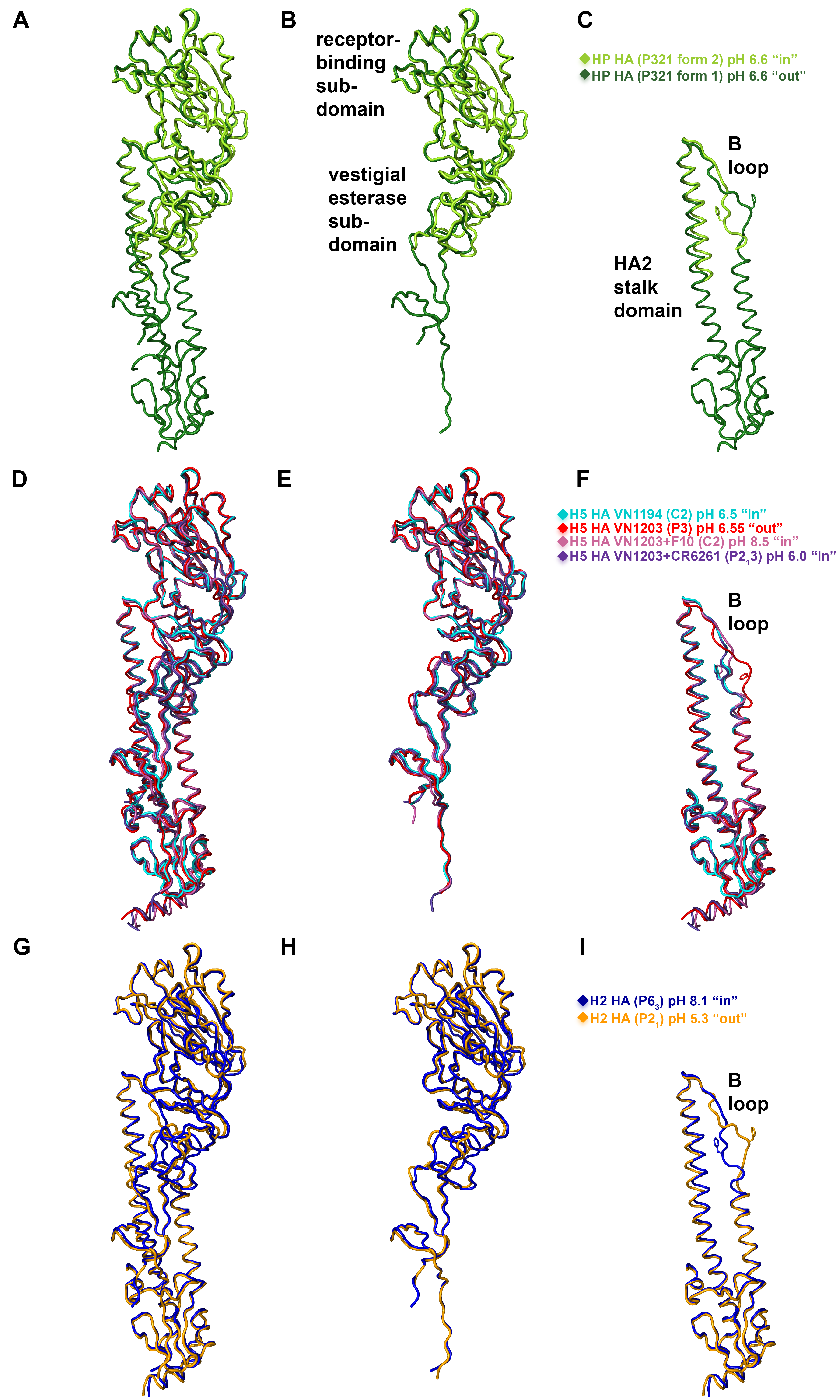

Supplement: Figure S5 — Comparison of HA structures. (A) Superposition of one protomer from the 2 crystal structures of HP HA. (B) Superposition of the HA1 chains from the 2 crystal structures of HP HA. (C) Superposition of the HA2 chains from the 2 crystal structures of HP HA. The variation between the interhelical B loops (“in” or “out” conformations) in the HP HA structures from two crystal forms at the same pH is likely the result of crystal packing differences. (D) Superposition of 1 protomer from four H5N1 HA crystal structures: VN1194 (PDB entry, 2IBX), VN1203 (PDB entry, 2FK0), VN1203 bound to antibody F10 (PDB entry 3FKU), and VN1203 bound to antibody CR6261 (PDB entry 3GBM). For clarification, the bound antibodies are not shown in the figure. (E) Superposition of the HA1 chains from the four H5N1 crystal structures in D. (F) Superposition of the HA2 chains from the four H5N1 crystal structures in D. (G) Superposition of one protomer from two H2 HA crystal structures. H2 HA (P63) corresponds to PDB entry 3QQB and H2 HA (P21) corresponds to PDB entry 3QQO. (H) Superposition of the HA1 chains from the two crystal structures of H2 HA. (I) Superposition of the HA2 chains from the two crystal structures of H2 HA. The crystallization space groups are described in parentheses; the crystallization pH is also indicated. (TIF) [file ppat.1002398.s005.tif]
